# Significant genetic differentiation among populations of *Anomalocardia brasiliana* (Gmelin, 1791): A bivalve with planktonic larval dispersion

**DOI:** 10.1590/S1415-47572009000200033

**Published:** 2009-06-01

**Authors:** Cinthya Cristina Bulhões Arruda, Colin Robert Beasley, Marcelo Vallinoto, Nelane do Socorro Marques-Silva, Claudia Helena Tagliaro

**Affiliations:** 1 Laboratório de Conservação e Biologia Evolutiva, Instituto de Estudos Costeiros, Campus Universitário de Bragança, Universidade Federal do Pará, Bragança, PABrazil; 2 Laboratório de Moluscos, Instituto de Estudos Costeiros, Campus Universitário de Bragança, Universidade Federal do Pará, Bragança, PABrazil; 3 Laboratório de Genética e Biologia Molecular, Instituto de Estudos Costeiros, Campus Universitário de Bragança, Universidade Federal do Pará, Bragança, PABrazil

**Keywords:** mtDNA, COI, population, *Anomalocardia brasiliana*, conservation

## Abstract

Four Brazilian populations of *Anomalocardia brasiliana* were tested for mutual genetic homogeneity, using data from 123 sequences of the mtDNA cytochrome oxidase c subunit I gene. A total of 36 haplotypes were identified, those shared being H3 (Canela Island, Prainha and Acupe) and both H5 and H9 (Prainha and Acupe). Haplotype diversity values were high, except for the Camurupim population, whereas nucleotide values were low in all the populations, except for that of Acupe. Only the Prainha population showed a deviation from neutrality and the SSD test did not reject the demographic expansion hypothesis. *Fst* values showed that the Prainha and Acupe populations represent a single stock, whereas in both the Canela Island and Camurupim stocks, population structures are different and independent. The observed structure at Canela Island may be due to the geographic distance between this population and the remainder. The Camurupim population does not share any haplotype with the remaining populations in northeastern Brazil. The apparent isolation could be due to the rocky barrier located facing the mouth of the Mamanguape River. The results highlight the importance of wide-scale studies to identify and conserve local genetic diversity, especially where migration is restricted.

## Introduction

The bivalve *Anomalocardia brasiliana* (Gmelin, 1791) is a venerid clam that occurs in the West Indies and South America from Surinam to Uruguay (Warmke and Abbott, 1961; Rios, 1994; Abbott and Morris, 1995). The popular name for this species in Brazil is “berbigão”, and in the French West Indies it is known as “chaubette” (Mouëza *et al.*, 1999). *A. brasiliana* is both consumed and commercialized by poor coastal communities, and is thus an important source of revenue (Mello and Tenório, 2000; Boehs and Magalhães, 2004; Barreira and Araújo, 2005).

*Anomalocardia brasiliana* has a planktonic larval phase (Schaeffer-Novelli, Y, PhD Thesis. Instituto de Biociências da Universidade Federal de São Paulo, 1976). Higher levels of dispersal are more common in species with this kind of larval phase than in those with direct development (Collin, 2001). Thus, the former are expected to have a higher gene flow, lower genetic divergence and, consequently, lower levels of population structure than the latter (Hoskin, 1997; Murray-Jones and Ayre, 1997; Collin, 2001)*.* Genetic differences among local populations generally depend upon the relationship between dispersal and survival (Ventura *et al.*, 2004).

In marine systems, geographic boundaries frequently occur due to currents and other limiting factors such as temperature and salinity (Collin, 2001). For marine molluscs, ocean currents seem to have an important influence on the overall population structure of a species, as well as the phylogeographic history of a region (Wares and Turner, 2003). The South-Equatorial current flows towards the northeastern Brazilian coast, at which point (9° S - 15° S) it splits into the northern Brazilian current (or Guyanas current) in the direction of the Guyanas, and the Brazil current, flowing south along the Brazilian coast (Cirano *et al.*, 2006).

Several population-genetics studies have been developed with marine bivalves from the Brazilian coast. Silva and Solé-Cava (1994) studied the levels of genetic variation and population structure in *A. brasiliana* specimens from seven localities along the east coast of Brazil by means of alloenzymatic systems. In their study, calculated levels of population structure were low, wherefore the authors suggested that the planktonic dispersal of *A. brasiliana* may be effective in minimizing the differentiation of allele frequencies over long distances. Oliveira *et al.* (2005), also using alloenzyme markers, studied five populations of *Mytella guyanensis* and three of *Mytella charruana*, and found that in both species, population genetic structure was not associated with the direction of oceanic currents or due to an isolation-by-distance model.

Partial sequences of the cytochome oxidase C subunit I gene (COI), from four populations of *Crassostrea rhizophorae* revealed intra-specific genetic homogeneity between the populations (unpublished data), indicating the occurrence of wide larval dispersion, in spite of the direction of ocean currents. Furthermore, five populations of *Crassostrea gasar* (= *C. brasiliana*) from Pará (00° 50' 41” S; 47° 07' 27” W) to Piauí states (02° 48' 45” S; 41° 48' 45” W) were found to belong to a single large stock (unpublished data). However, studies on *Mytella guyanensis*, also with the use of partial COI gene sequences, showed a clear structure level for the Camurupim population (Paraíba state) when compared with populations from the North and East of Brazil, thus implying that local environmental factors may be influencing larval dispersal (unpublished data). The Camurupim estuary is surrounded by reefs (Alves and Nishida, 2002), these acting as a barrier that may reduce migration in certain species. On the other hand, the northern coast of Brazil is under the influence of riverine discharge, whereby at Canela Island there is a reduction in salinity during the rainy season (C. R. Beasley, unpublished data), this possibly being the cause of increased mortality in planktonic larval stages.

The aim of the present study is to genetically characterize four populations of *Anomalocardia brasiliana*, using COI partial sequences of mitochondrial DNA. The COI gene has already been used to study mollusk phylogeography (Hoeh *et al.*, 1997; King *et al.*, 1999), as well as to characterize populations and evaluate mutual gene flow (Shi *et al.*, 2002). The correct identification and molecular characterization of bivalve stocks are both essential for their management, in order to maintain genetic potential for future evolutionary change.

**Figure 1:**
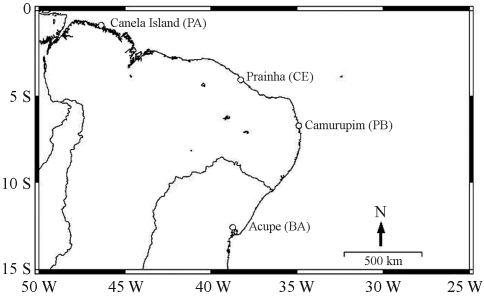
North and northeast Brazil showing the sampling sites.

**Figure 2:**
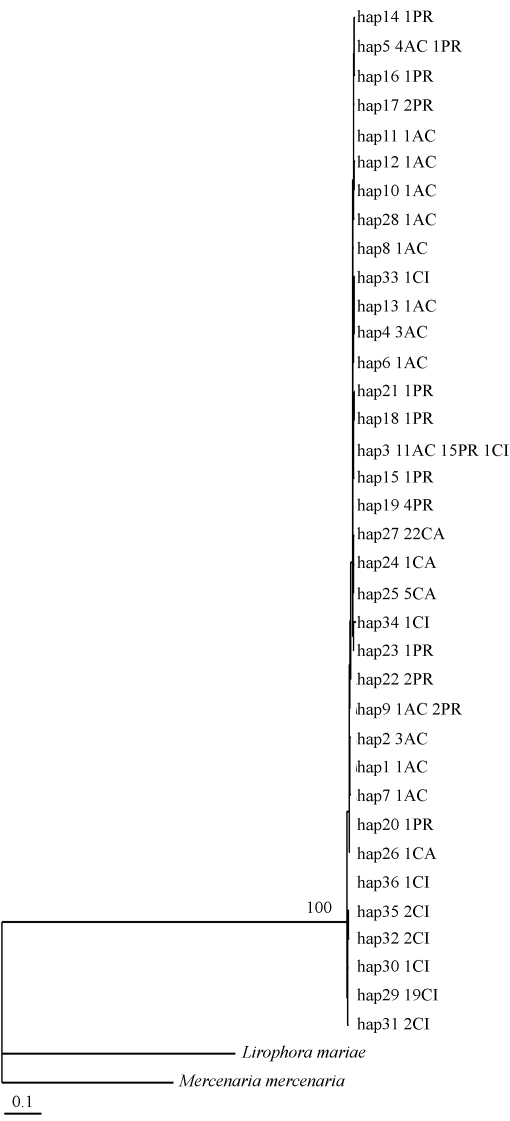
Maximum likelihood tree obtained from the present analysis of the 36 COI haplotypes of *Anomalocardia brasiliana*. The number above the branch indicates the only bootstrap value greater than 70%. CA = Camurupim; CI = Canela Island; PR = Prainha; AC = Acupe. Values preceeding abbreviations represent the number of individuals typed for each haplotype.

**Figure 3:**
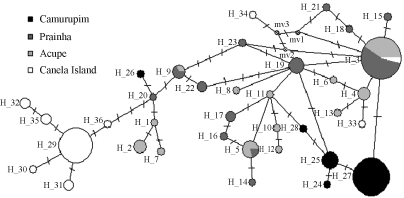
Median joining haplotype network. Each trace corresponds to a single mutation. The haplotypes are represented by circles, the width being proportional to their frequencies; mv1, mv2 and mv3 are median vectors.

## Materials and Methods

### Sampling

Specimens of *Anomalocardia brasiliana* were collected from Canela Island (00° 47' S, 46° 43' W; N = 30), in Pará State, northern Brazil and from three other localities in northeastern Brazil: Prainha (03° 53' S, 38° 21' W; N = 33), in Ceará State; Camurupim (06° 44' S, 34° 56' W; N = 30), in Paraíba State; and Acupe (12° 35' S, 38° 41' W; N = 30), in Bahia State (Figure 1).

### DNA extraction, PCR amplification and sequencing

The bivalve adductor muscle was removed and preserved in 100% ethanol and kept at -20 °C until DNA extraction. Total genomic DNA was isolated using the phenol-chloroform protocol of Sambrook *et al.* (1989). DNA amplification of partial mitochondrial COI gene sequences was obtained with the LCOCI1490 and HCOCI2198 primers designed by Folmer *et al.* (1994). The amplification reaction was carried out under the following conditions: initial denaturing at 95 °C for 3 min; 35 cycles of 95 °C denaturing for 1 min; 45 °C annealing for 1 min; 72 °C extension for 1.5 min; and a final extension at 72 °C for 7 min during the last cycle. PCR products were purified by using ExoSap IT enzymes (Amersham-Pharmacia Biotech. Inc., Piscataway, NJ, USA). Sequencing was performed on Mega Bace 750 and ABI 377 automated sequencers, according to manufacturer's protocols.

### Data analysis

Sequence alignment was undertaken with the Clustal X version 1.8 (Thompson *et al.*, 1997) with default parameters. Minor modifications were made using the BioEdit version 5.0.6 sequence editor (Hall, 1999). Nucleotide sequence data for the haplotypes used in this paper were deposited in GenBank under accession numbers FJ481182 - FJ481217. Mega version 3.1 (Kumar *et al.*, 2004) was used to obtain nucleotide frequencies and the average transition/transversion ratio, as well as in checking for termination codons. A saturation test was performed using the DAMBE program (Xia and Xie, 2001).

Phylogenetic analyzes to generate the maximum likelihood (ML) tree were performed with the PHYML version 2.4.4 (Guindon and Gascuel 2003), with gaps being treated as missing data. The evolutionary model was selected using MODELTEST, version 3.07 (Posada and Crandall, 1998), and adopting the AIC criterion (Posada and Buckley, 2004) for the ML tree and distance matrix. The ML tree was generated by using a single sequence of each haplotype, and as outgroups, one sequence of *Mercenaria mercenaria* (GenBank EU360429) and one of *Lirophora mariae* (GenBank DQ458483). The evaluation of statistical confidence was based on bootstrapping with 1,000 pseudo-replicates for ML (Felsenstein, 1985). The criterion adopted for evaluating robustness was to consider nodes with bootstrap values equal or superior to 90% as being well supported. PAUP* version 4.0 (Swofford, 2003) was used to generate the distance matrix, using all 123 sequences.

DnaSP version 4.10 (Rozas *et al.*, 2003) was used to identify haplotypes and to run the McDonald and Kreitman Test (McDonald and Kreitman, 1991). This test is based on a comparison of synonymous and non-synonymous (replacement) variation within and between species. Under neutrality, the ratio of replacement to synonymous fixed substitutions (differences) between species should be the same as the ratio of replacement to synonymous polymorphisms within species. Network version 4.5.0.0 (Bandelt *et al.*, 1999) was used to draw the haplotype network calculated by using the median-joining option.

Under the assumption of selective neutrality, and through mismatch distribution analyses, possible historical events in population growth and decline were evaluated (Rogers and Harpending, 1992). Theoretical distributions based on sudden expansion model assumptions were compared to observed data. The goodness-of-fit of observed data to a simulated expansion model was tested with the sum of squared deviations (SSD) using Arlequin 3.1 (Excoffier *et al.*, 2005) with 10,000 bootstrap replicates (Slatkin and Hudson, 1991; Rogers and Harpending, 1992). This program was also used to verify haplotype (*h*; Nei, 1987) and nucleotide (π; Nei, 1987) genetic diversity indices, to execute Fu's *Fs* test (Fu, 1997) with 10,000 simulated samples, to ascertain possible neutrality deviations and to infer population dynamics. We used Grant and Bowen's (1998) criteria on considering values of *h* and π inferior to 0.5 and 0.005, respectively, as being small. Pairwise genetic divergence between populations was estimated by using *Fst* values (Excoffier *et al.*, 1992), and significance was verified through 10,000 permutations. Partitioning of genetic variability among populations was tested by means of hierarchical analysis of molecular variance AMOVA (Excoffier *et al.*, 1992), by considering one group with four populations and using Arlequin version 3.01. Both analyses were carried out with 10,000 permutations.

## Results

The COI amplicons were approximately 650 bp long. One hundred and twenty three unambiguously aligned sequences from four populations were obtained for 580 bp of the COI coding sequence from *A. brasiliana,* of which 19 sites were variable and all parsimony informative. The transition/transversion rate was 6.9 and the nucleotide composition of COI sequences was 41.0% (thymine), 14.8% (cytosine), 23.3% (adenine) and 20.9% (guanine). There were no indels and no termination codons. There was no evidence of saturation. Amongst the four populations studied, a total of 36 haplotypes were identified for the COI gene.

The maximum likelihood best fit model selected by AIC for the 36 *A. brasiliana* haplotypes and the *Mercenaria mercenaria* (GenBank EU360429) and *Lirophora mariae* (GenBank DQ458483) sequences used to generate the ML tree were: base frequencies (A = 0.2349, C = 0.1280, G = 0.2079, T = 0.4292), gamma distribution shape parameter (α = 1.3625) and substitution model rate matrix (Rmat; A-C = 26.6092, A-G = 40.8919, A-T = 0.0000, C-G = 11.2906, C-T = 195.1375, G-T = 1.0000), the proportion of invariable sites being 0.6548. The rooted ML tree showed weak bootstrap values not supporting geographical haplotype groups (Figure 2). The maximum likelihood best fit model selected by AIC for the 123 *A. brasiliana* sequences used to obtain the distance matrix, were base frequencies (A = 0.2284, C = 0.1553, G = 0.2076, T = 0.4086), gamma distribution shape parameter (α = 0.4630) and substitution model rate matrix (Rmat; A-C = 5.9126 A-G = 6.3174, A-T = 0.0000, C-G = 9.2038, C-T = 43.3821, G-T = 1.0000), the proportion of invariable sites being 0.8999. The distance matrix based on the General Time-Reversible model (GTR; Lanave *et al.*, 1984; Rodriguez *et al.*, 1990) showed low divergence values between samples of the four populations (d = 0 to 0.021), these being compatible with intra-specific values.

In the population analysis, the number of observed haplotypes within populations were 9 on Canela Island, 13 at Prainha, 5 at Camurupim and 13 at Acupe (Table 1). The shared haplotypes among populations were H3 (Canela Island, Prainha, Acupe), and H5 and H9 (Prainha and Acupe). The Camurupim population did not share any haplotypes with the others, the most frequent haplotype in this population (H27) showing only one mutation (site 15) when compared to H3, found in all the other populations. Considering only the most frequent haplotypes (H3, H27 and H29), H29, encountered only on Canela Island, was the most distinct haplotype revealing eight mutations in relation to H3, the most frequent at Prainha and Acupe, and nine in relation to H27, the most common at Camurupim. The intrapopulation number of mutations was 1 to 6 at Camurupim, 1 to 8 at Prainha, 1 to 9 at Acupe and 1 to 10 on Canela Island.

In the haplotype network (Figure 3), the separation of the Canela Island haplotypes was evident, except for H33 and H34, which probably arrived at the Pará coast from northeastern Brazil via the North Brazil current. On the other hand, the haplotypes found in the Camurupim population showed high similarity with those found in other northeastern Brazilian populations. Haplotype diversity values were high, except for the Camurupim population, whereas nucleotide values were low in all populations, except for Acupe (Table 2). Fu's Neutrality *Fs* test (Fu, 1997) was significant only for the Prainha population and results from the SSD test did not reject the demographic expansion hypothesis (Table 2). The McDonald and Kreitman Test (McDonald and Kreitman,1991) showed no significant differences between the four *A. brasiliana* populations. The ratio of replacement to synonymous fixed substitutions between these populations were the same as the ratio of replacement to synonymous polymorphisms within each of them. On the other hand, comparison of the substitution ratio of *A. brasiliana* populations from Camurupim and Canela Island with *Mytella guyanensis* (unpublished data), from the same geographic region, revealed significant differences between the substitutions of the two species from Canela Island (Neutrality Index = 0,314; α = 0,686; Fisher's exact test. P-value - two tailed: 0,004374). However, most nucleotide mutations of *A. brasiliana* sequences from Canela Island were synonymous (N = 25), and only those found in haplotypes H32 and H33 were exclusive for that population (Table 1).

*Fst* probability (P) values showed that the Prainha and Acupe populations were not significantly different from one another (Fst = 0.034; p = 0.077). *Fst* values of the other populations ranged from 0.366 (Camurupim and Acupe) to 0.813 (Canela Island and Camurupim), all being highly significant (Table 3). The AMOVA results for a single group of four populations (Table 4) indicated that a greater part of the variation was among populations (variation: 57.63%; p < 0.001).

## Discussion

The larval stage in *Anomalocardia brasiliana* is planktonic, and studies of allozymes and mitochondrial DNA have shown that fish and invertebrate species with planktotrophic larvae are genetically similar over wide regions, though not necessarily throughout their whole range (Hedgecock, 1994, Oliveira *et al.*, 2005). Migration between populations is a potent and systematic means of homogenizing the gene pools of conspecific populations (Hedgecock, 1994), and the genetic differentiation observed among populations of *A. brasiliana* may often be explained by a combination of several factors, including local environmental features (temperature, salinity, physical barriers, etc.), geographic distances and the direction of marine currents.

On considering the latitude of forking in the South-Equatorial marine current (Cirano *et al.*, 2006), homogeneity had already been expected among the Canela Island, Prainha, Camurupim and Acupe populations. Our results showed that differences exist among the populations, except for those at Acupe and Prainha. Significant differences between Camurupim and three other Brazilian populations of *Mytella guyanensis* (Mollusca, Bivalvia) have already been observed (unpublished data).

Moreover, in the *A. brasiliana* population from Camurupim, there is no haplotype in common with those in the other populations, and thus seems to be heading for isolation. The most frequent haplotype from Camurupim differs in only one mutation from the most common one present in Prainha and Acupe. The similarity between the two suggests that in the past there may have been a founder effect in Camurupim, followed by a punctual mutation. Alternatively, the most frequent allele at Camurupim may be present in the other populations but at low frequencies, although this was not detected through our sampling. Moreover, according to Grant and Bowen's (1998) criteria, the low haplotype and nucleotide diversities found in this population suggest that it may have undergone a recent bottleneck or founder event.

The present isolation of the Camurupim population may be partially explained by the presence of a rocky barrier located at the mouth of the Mamanguape River (see Alves and Nishida, 2002). Beachrocks from the Brazilian northeast have been dated using the radiocarbon method, the maximum estimated age being 7,460 calendar years before the present (Bezerra *et al.*, 2003). According to Bilton *et al.* (2002), the degree of larval retention in estuarine areas is partially dictated by local characteristics, such as the amount and type of water exchange in the estuary. Hence, the presence of a rocky barrier that may limit water exchange in Camurupim might explain the lack of gene flow between this population and the others.

The bifurcation in the South-Equatorial current (9° S -15° S) generates the North Brazilian current (Cirano *et al.*, 2006), which would be expected to cause the distribution of *A. brasiliana* larvae to become homogenous throughout the northern Brazilian coast. On the other hand, it could be expected that the pronounced coastal environmental differences, due to the enormous discharge of fresh water and sediments from Amazonian rivers during the rainy season, could put pressure on the population, selecting for particular alleles. However, this does not seem to be the case in COI, in spite of the great differences observed between nucleotide sequences from Canela Island, where few differences were observed in the resulting amino acid sequences. Moreover, salinity on Canela Island ranged from 20 to 40 between August 2001 and December 2003 (C. R. Beasley, unpublished data), being similar to that observed in other studies on this species (Schaeffer-Noveli Y, PhD Dissertation, Universidade de São Paulo, São Paulo, 1976; Mouëza *et al.*, 1999; Arruda and Amaral, 2003). However, *Fst* values and AMOVA showed that the Canela Island population was not homogeneous with the other three populations, probably due to the greater geographic distances or to hitchhiking.

According to Crandall *et al.* (2000), genetic techniques provide estimates of gene flow between populations, and thus guide efforts to maintaining historical levels of genetic exchange between populations. In the present study, the Camurupim population was shown to be at least partially isolated, as was the *M. guyanensis* population, also from the same location (unpublished data). These results indicate that this region should be studied in detail in order to verify whether this pattern of isolation exists for other aquatic species from the Mamanguape estuary. The COI molecular studies of Luttikhuizen *et al.* (2003) and the research of Cho *et al.* (2007) presented similar results. Luttikhuizen *et al.* (2003) detected signs of population divergence in *Macoma balthica* in Europe, that appears to have originated during the Pleistocene, some of the current populations appearing to be connected by gene flow whereas others are isolated. On the other hand, Cho *et al.* (2007), on studying populations of *Scapharca roughtonii* from Korea, also found differences between the population from Jinhae and six other Korean populations. The authors state that the presence of a narrow avenue for exchange with offshore waters affords limited dispersal. According to Luttikhuizen *et al.* (2003), some bivalve populations can remain highly subdivided in spite of the potential for high gene flow, this implying that their population and evolutionary dynamics can be independent.

The observed partial isolation of the Camurupim population highlights the importance of wide-ranging conservation programs involving several different regions, in order to preserve local genetic diversity, especially if migration is partially or completely restricted.

## Figures and Tables

**Table 1:** COI gene haplotypes from samples of *Anomalocardia brasiliana* showing the variable nucleotide and amino acid sites, and the number of individuals carrying each haplotype.

Haplotypes	Variable nucleotide sites	Variable amino acids and corresponding nucleotide mutated sites	Acupe	Prainha	Camurupim	Canela Island
	1111123 344445555	445				
	1571112572 226890113	691				
	5424780301 717059074	750				
H1	GAGTTCACTT CTCCGTACT	ALM	1	0	0	0
H2	.......... ......C..	..I	3	0	0	0
H3	..ACC...CC .CT......	V..	11	15	0	1
H4	..ACC...CC .CT...C..	V.I	3	0	0	0
H5	...CC...CC .CTT...T.	V..	4	1	0	0
H6	...CC...CC .CT...C..	V.I	1	0	0	0
H7	.........C .........	...	1	0	0	0
H8	...CC...CC .C.......	...	1	0	0	0
H9	.......... .CT......	V..	1	2	0	0
H10	...CC...CC .CTTT....	VF.	1	0	0	0
H11	...CC...CC .CTT.....	V..	1	0	0	0
H12	...CC...CC .C.TT....	.F.	1	0	0	0
H13	..ACC...CC .C....C..	..I	1	0	0	0
H14	...CCT..CC .CTT...T.	V..	0	1	0	0
H15	..ACC...CC .CT....T.	V..	0	1	0	0
H16	...CC...CC TCTT...T.	V..	0	1	0	0
H17	...CC...CC TCTT.....	V..	0	2	0	0
H18	..ACC.G.CC. CT......	V..	0	1	0	0
H19	...CC...CC. CT......	V..	0	4	0	0
H20	.......... ..T......	V..	0	1	0	0
H21	..ACC.G.CC TCT......	V..	0	1	0	0
H22	.........C .CT......	V..	0	2	0	0
H23	...CC...C. .CT......	V..	0	1	0	0
H24	C..CC...CC TCT......	V..	0	0	1	0
H25	C..CC...CC .CT......	V..	0	0	5	0
H26	C......... ..T......	V..	0	0	1	0
H27	C.ACC...CC .CT......	V..	0	0	22	0
H28	C..CC...CC .CTT.....	V..	0	0	1	0
H29	.G.....T.C ..T.....C	V..	0	0	0	19
H30	.G...T.T.C ..T.....C	V..	0	0	0	1
H31	.G.....T.C T.T.....C	V..	0	0	0	2
H32	.G.....T.C ..T..CC.C	V.T*	0	0	0	2
H33	..ACC...CC .CT..CC..	V.T*	0	0	0	1
H34	..ACC...C. T.T......	V..	0	0	0	1
H35	.G.....T.C ..T...C.C	V.I	0	0	0	2
H36	.G.....T... .T.....C	V..	0	0	0	1

Total			30	33	30	30

*Also mutated in the 2^nd^ position of the codon (nucleotide site 509).

**Table 2:** Cytochrome oxidase c subunit I (COI) diversity, mutation neutrality test results and mismatch analysis for each population.

Populations	N	H	*h*	π	*Fs*	SSD
Acupe BA	30	13	0.8460	0.0067	-3.19460 (p = 0.0797)	0.0119 (p = 0.7903)
Prainha CE	33	13	0.7841	0.0042	-4.99549 (p = 0.0103)	0.0047 (p = 0.8865)
Camurupim PB	30	5	0.4460	0.0015	-0.99193 (p = 0.2424)	0.0044 (p = 0.3771)
Canela Island PA	30	9	0.6000	0.0039	-1.66174 (p = 0.2059)	0.0156 (p = 0.5038)

N = sample size; H = number of haplotypes; *h* = haplotype diversity; π = nucleotide diversity; Fs = Fu's Fs; SSD = sum of squared deviations.

**Table 3:** Fixation indices *Fst* (below diagonal), inter-population average distance matrix according to the Tamura-Nei model (above diagonal) and intra-population distance values (diagonal and bold).

	Acupe	Prainha	Camurupim	Canela Island
Acupe	**0.0064**	0.0055	0.0062	0.0136
Prainha	0.0340	**0.0043**	0.0048	0.0132
Camurupim	0.3667*	0.3852*	**0.0015**	0.0149
Canela Island	0.6155*	0.6829*	0.8135*	**0.0040**

*p < 0.001

**Table 4:** AMOVA results considering one group with four populations (Canela Island, Prainha, Camurupim and Acupe populations).

Source of variation	D.f.*	Sum of squares	Variance components	Percentage of variance	Fixation indices	Probability (p)
Among populations	3	148.864	1.57693	57.63	F_ST_ = 0.57627	0.000**
Within populations	119	137.982	1.15951	42.37	-	-

Total	122	286.846	2.73644			

*D.f.: degrees of freedom; **p < 0.001.
